# Dynamic evolution of the anterior cingulate‐insula network during seizures

**DOI:** 10.1111/cns.14310

**Published:** 2023-06-12

**Authors:** Yujiao Yang, Dong Chen, Jing Wang, Jie Wang, Zhaofen Yan, Qinqin Deng, Liping Zhang, Guoming Luan, Mengyang Wang, Tianfu Li

**Affiliations:** ^1^ Department of Neurology, Sanbo Brain Hospital Capital Medical University Beijing China; ^2^ Key Laboratory of Mental Health Institute of Psychology, Chinese Academy of Sciences Beijing China; ^3^ Department of Electrophysiology Capital Institute of Pediatrics Beijing China; ^4^ Department of Functional Neurosurgery, Sanbo Brain Hospital Capital Medical University Beijing China; ^5^ Beijing Key Laboratory of Epilepsy, Sanbo Brain Hospital Capital Medical University Beijing China; ^6^ Beijing Institute for Brain Disorders, Capital Medical University Beijing China

**Keywords:** anterior cingulate cortex, epilepsy, excitation–inhibition ratio, functional connectivity, insula

## Abstract

**Objectives:**

In physiological situations, the anterior cingulate cortex (ACC) and anterior insular cortex (AIC) are prone to coactivation. The functional connectivity and interaction between ACC and AIC in the context of epilepsy remain unclear. This study aimed to investigate the dynamic coupling between these two brain regions during seizures.

**Methods:**

Patients who underwent stereoelectroencephalography (SEEG) recording were included in this study. The SEEG data were visually inspected and quantitatively analyzed. The narrowband oscillations and aperiodic components at seizure onset were parameterized. The frequency‐specific non‐linear correlation analysis was applied to the functional connectivity. The excitation/inhibition ratio (E:I ratio) reflected by the aperiodic slope was performed to evaluate the excitability.

**Results:**

Twenty patients were included in the study, with 10 diagnosed with anterior cingulate epilepsy and 10 with anterior insular epilepsy. In both types of epilepsy, the correlation coefficient (*h* ^2^) between the ACC and AIC at seizure onset exhibited a significantly higher value than that during interictal and preictal periods (*p* < 0.05). The direction index (D) showed a significant increase at seizure onset, serving as an indicator for the direction of information flow between these two brain regions with up to 90% accuracy. The E:I ratio increased significantly at seizure onset, with the seizure‐onset zone (SOZ) demonstrating a more pronounced increase compared to non‐SOZ (*p* < 0.05). For seizures originating from AIC, the E:I ratio was significantly higher in the AIC than in the ACC (*p* = 0.0364).

**Conclusions:**

In the context of epilepsy, the ACC and AIC are dynamically coupled during seizures. The functional connectivity and excitability exhibit a significant increase at seizure onset. By analyzing connectivity and excitability, the SOZ in ACC and AIC can be identified. The direction index (D) serves as an indicator for the direction of information flow from SOZ to non‐SOZ. Notably, the excitability of SOZ changes more significantly than that of non‐SOZ.

## Graphical abstract

The ACC and AIC are dynamically coupled during seizures. The functional connectivity between ACC and AIC increases significantly at seizure onset. The E:I ratio changed dynamically during seizures and can be used to identify the brain region which possesses higher epileptogenicity.

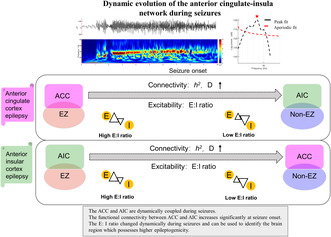

## 1 INTRODUCTION

Recent evidence suggests that the anterior cingulate cortex (ACC) and anterior insular cortex (AIC) are susceptible to coactivation in physiological situations. When human brains detect salient events, integrate stimuli to cognitive control, and respond appropriately in behavior, the ACC and AIC are coactivated.^1^, ^2^, ^3^ Cytologically, ACC and AIC have a common basis for coactivation. They both have Von Economo Neurons (VENs) cells in layer 5, and the main function of this cell is to monitor external stimuli.^4^, ^5^ In terms of cellular architecture, ACC and AIC are homogeneous in that they both lack a granular layer.^6^, ^7^ Studies on structural connectivity have also confirmed a direct fiber connection between these two brain regions.^8^ We observed that in some patients with drug‐resistant epilepsy, the ACC and AIC exhibited synchronous onset on stereoelectroencephalography (SEEG) due to early propagation, making visual distinction challenging. Therefore, we are interested in how ACC and AIC interact in the epileptic process.

Epilepsy is fundamentally a network disease. The concept of epileptogenic networks (ENs) opens a window to understanding epileptogenic processes.^9^ The EN (including seizure generation and propagation) is responsible for the clinical and electrographic phenomena.^10^, ^11^ Functional connectivity (FC) studies can deliver unique insight into how functional communication is implemented between different brain regions in ENs.^12^ When performing FC analysis, the SEEG signal is superior to neuroimaging signal because it can be performed over a broad range of time scales. Non‐linear correlation analysis gives an estimate of the degree and directionality of functional coupling between brain regions.^13^ The degree of FC during different periods of seizure might provide a better understanding of the dynamic functional coupling of ACC and AIC at seizure onset. The directionality of FC could reveal the leader–follower relationship between these two regions, which may assist in identifying the brain region with higher epileptogenicity.

Epilepsy is considered a network‐level dysfunction between excitation (E) and inhibition (I). The transition from interictal to ictal may be caused by multiple mechanisms and a combination of causally linked cascading events.^14^ The combined effect of these events results in an E/I imbalance, which is the major determinant of brain excitability.^10^, ^14^ Existing methods for estimating the excitation/inhibition ratio (E:I ratio) are limited to small cell populations, making them difficult to apply clinically. Fortunately, at the population level, the local field potential (LFP) that can be recorded with SEEG is contributed mostly by synaptic activity.^15^ The synaptic input can be modeled by summing two stationary stochastic processes representing excitatory and inhibitory inputs.^16^ Therefore, we are able to obtain the E:I ratio by measuring the LFP. A consistent quantitative feature of the LFP is the magnitude of LFP power which is inversely related to temporal frequency.^17^ The slope of power spectrum density (PSD) refers to the rate of decline of PSD as a function of frequency in the aperiodic range. This parameter takes into consideration the power across the entire frequency spectrum and reflects the E:I ratio of the underlying neuronal population.^18^ Therefore, we applied the PSD slope to evaluate the dynamic evolution of the E:I ratio of both the seizure‐onset zone (SOZ) and non‐SOZ.

In this study, both FC based on periodic oscillations and aperiodic slopes of the SEEG signal were used. In order to address our hypothesis that there exists a dynamic coupling between the ACC and AIC during seizures, we tried to explore: (1) whether there exits FC between ACC and AIC during seizures; (2) how the ACC and AIC interact during seizure‐onset periods. To identify the SOZ in ACC and AIC, we tried to investigate: (1) whether there is a leader–follower relationship in FC between the SOZ and non‐SOZ during seizures; (2) how the excitability of both SOZ and non‐SOZ changes during seizures.

## 2 MATERIALS AND METHODS

**FIGURE 1:**
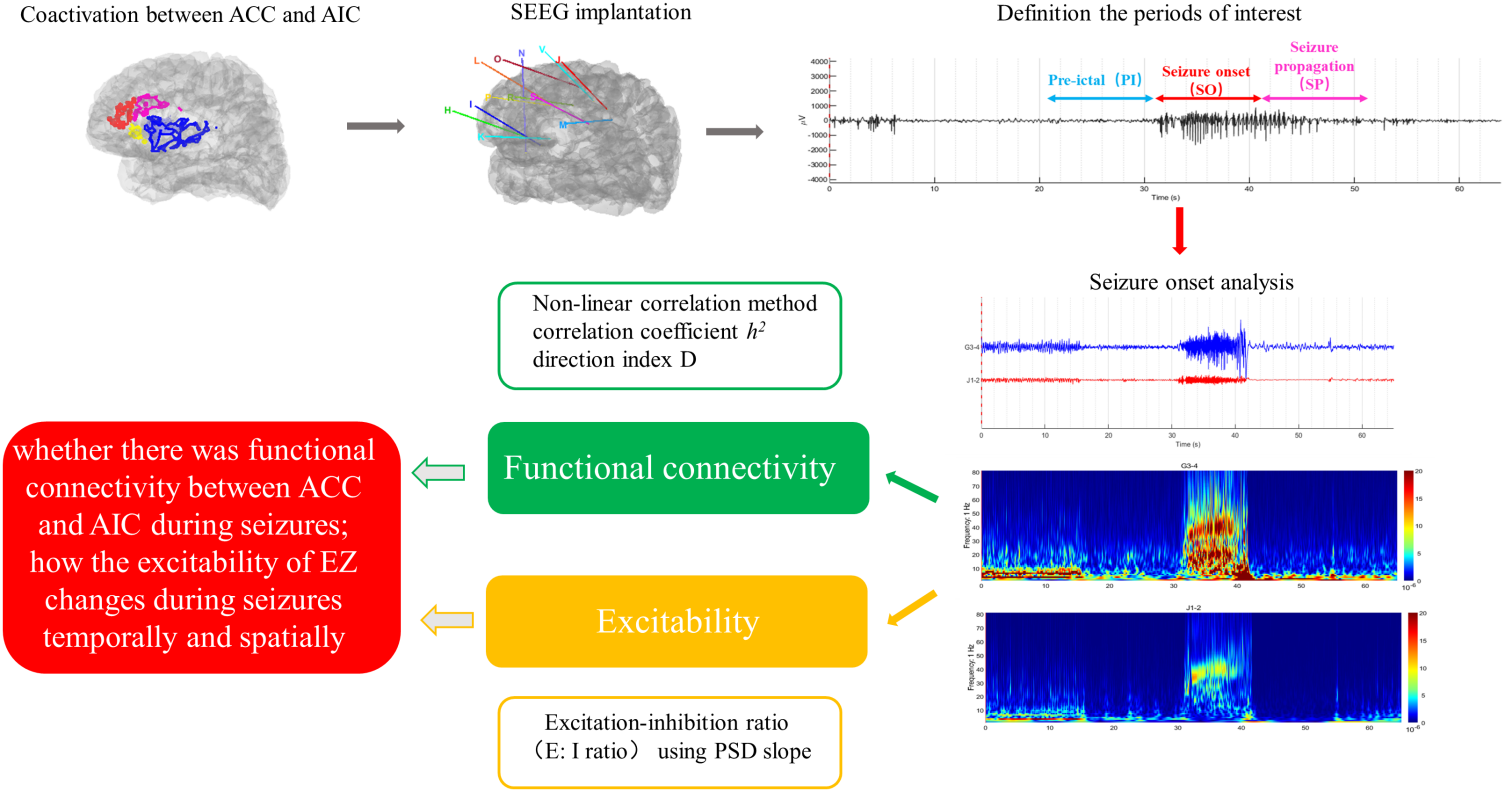
Summary of the analysis pipeline. Coactivation is a specific phenomenon. Patients who underwent intracranial electrode implantation were enrolled. The period of interest was defined by visual analysis. The frequency of interest was defined by time‐frequency analysis. The functional connectivity and excitability were used to explore the reasons for coactivation between ACC and AIC and to investigate the methods to distinguish which is the SOZ in ACC and AIC. ACC, anterior cingulate cortex; AIC, anterior insula cortex; SEEG: stereoelectroencephalography; *h* ^2^: non‐linear correlation coefficient; *D*, direction index.

Figure 1 summarizes the methods that were used in the present study.

### 2.1 Patient selection

Patients with medically intractable epilepsy who underwent SEEG evaluation at the Sanbo Brain Hospital from January 2015 to December 2020 were retrospectively analyzed in this study. The inclusion criteria were as follows: (i) anterior cingulate gyrus epilepsy or anterior insula epilepsy defined by SEEG; (ii) at least one intracerebral electrode contact implanted in each of the ACC and AIC; (iii) surgical resection or radiofrequency thermocoagulation (RF‐TC) after SEEG monitoring; and (iv) seizure free or a significant reduction in seizures after treatment (Engel I or II).^19^ Patients with inadequate postoperative follow‐up (less than 1 year or lost visits) or SEEG with continuous artifacts were excluded.

In this study, all patients underwent routine preoperative evaluation, including scalp video‐EEG monitoring, magnetic resonance imaging (MRI), positron emission tomography (PET), magnetoencephalography (MEG), and neuropsychological assessment. Patients with conflicting clinical, electrical, and/or imaging data were discussed at the Epilepsy Center Conference, and SEEG implantation was performed for preoperative evaluation. The surgical implantation of electrodes was performed based on presurgical anatomic‐electroclinical hypotheses. Intracerebral multiple‐contact electrodes (8–16 contacts; length: 2 mm, diameter: 0.8 mm at 1.5 mm apart; Huake‐Hengsheng Medical Technology Co. Ltd.) were implanted with a robot‐assisted stereotactic surgery system. SEEG recordings were obtained with a Nicolet system (128 channels, sampling rate: 512 Hz or 256 Hz; Thermo Nicolet Corporation). A postoperative computerized tomography (CT) scan was used to verify the absence of bleeding and the position of electrodes. SEEG recordings were performed as necessary (1–3 weeks) to record several habitual seizures in the patients.

Surgical resection or RF‐TC therapy was performed according to the results of the invasive evaluation. Histopathological examination of tissue specimens was also performed. Surgical outcomes were regularly assessed during postoperative follow‐up and documented according to Engel's classification.

The institutional review board of the Ethics Committee of Sanbo Brain Hospital, Capital Medical University approved this study, and a written patient consent was obtained from all included patients.

### 2.2 Definition of regions of interest

We analyzed the bipolar signals recorded from two regions of interest (ACC and AIC). CT‐scan/MRI data fusion was performed, and reconstructed images were digitally fused with the presurgical MRI dataset. The resulting merged datasets were displayed and reviewed in three orientation planes to determine the precise anatomical location of the electrodes. Based on the three‐dimensional fusion of preoperative MRI and postoperative CT, the two regions were labeled using Automated Anatomical Labeling atlas 3 (AAL3), and the corresponding electrode contacts were identified.

### 2.3 SEEG analysis

#### 2.3.1 Visual analysis and definite periods of interest

One typical habitual seizure in each patient was analyzed visually. More than one seizure was recorded in all patients, and one of the seizures was randomly chosen to avoid selection bias. All seizures of patients were analyzed by two independent epileptologists blinded to the clinical data, and discrepancies were resolved by a senior epileptologist. The time of seizure onset was determined cautiously. The seizure onset was indicated by a variety of stereotypical electrographic features, which include, but were not limited to, low‐voltage fast activity, low‐frequency high‐amplitude periodic spikes, sharp activity at ≤13 Hz, spike‐and‐wave activity, burst of high‐amplitude polyspikes, or burst suppression.^20^ The SOZ determination occurred during the intracranial recording session and was completed before data analysis in this study.

To quantify the dynamic changes between the ACC and AIC from interictal to ictal, we chose four periods of interest (10 s each): (i) interictal period (IID): 10 s at least 2 h before seizure onset; (ii) preictal (PI): 10 s before seizure onset; (iii) seizure onset (SO): 10 s after seizure onset; and (iv) seizure propagation (SP): 10–20 s after seizure onset.

#### 2.3.2 Quantitative Analysis and definite frequency of interest

SEEG data were analyzed using the Brainstorm toolbox for MATLAB.^21^ The power spectrum density (PSD) was evaluated using Welch's method (10 s time window, 0.5 s window length, 50% overlap). The method, “fitting oscillations and one‐over‐F” (FOOOF), was performed on Welch's PSD files to decompose the SEEG signals into periodic and aperiodic components.^22^ Using this model‐driven approach, the FOOOF algorithm can extract both periodic and aperiodic elements within the overall power spectrum. In this study, we extracted the center frequency and bandwidth for the oscillatory components of the signal. The aperiodic slope across a broad frequency range between 1 and 80 Hz was obtained. The peak model was established using a Gaussian distribution. The spectral parameterization settings for the algorithm were as follows: peak width limits = [0.5, 12], maximum number of peaks = 3, and aperiodic mode = fixed.

The central frequencies and bandwidths during seizures were extracted from the periodic signals. For statistical purposes, all frequency bands were divided into seven groups: *δ* (0.5–4 Hz), *θ* (4–8 Hz), *α* (8–13 Hz), low *β* (13–20 Hz), high *β* (20–30 Hz), low *γ* (30–45 Hz), and high *γ* (45–80 Hz). The bands during seizures were individualized for each patient. A unique frequency band was selected for each patient. Functional connectivity analysis was based on frequency‐specific sub‐bands.

### 2.4 Frequency‐specific FC analysis

The FC was estimated using a non‐linear nonparametric regression between the ACC and AIC.^13^ This computational approach was applied to the analysis of SEEG signals in the context of epilepsy.^9^ This method gives an estimate of the degree of functional coupling using the correlation coefficient *h* ^2^ and directionality using direction index (D). The *h* ^2^ is a nonparametric analysis aimed at quantifying the amplitude correlation of signal *Y* on signal *X*. The *h* ^2^ is bounded between 0 (no correlation) and 1 (maximal correlation). The values of $h_{X\to Y}^{2}$ and $h_{Y\to X}^{2}$ were asymmetric, as shown in equation (1).

(1) $$h_{\mathrm{XY}}^{2}\left(\tau \right)=1-\frac{\mathrm{VAR}\left[Y\left(t+\tau \right)/X\left(t\right)\right]}{\mathrm{VAR}\left[Y\left(t+\tau \right)\right]}$$

In addition, we also defined the time lag *t* between *X* and *Y* using the asymmetry. The time lag *t* was used to estimate the time delay between two signals. Consequently, by using time delay and asymmetry information for each pair of structures, the direction index (*D*) can be calculated for each period.^23^ To detect the information flow between ACC and AIC, we calculated the direction index (*D*), as shown in equation (2):

(2) $$D_{\mathrm{xy}}=\frac{1}{2}\left[\mathrm{sgn}\left(\Delta h^{2}\right)+\mathrm{sgn}\left(\Delta t\right)\right]$$

If *D*_*xy*_ = +1, *Y* is influenced by *X*; if *D*_*xy*_ = −1, *X* is influenced by *Y*. In the present study, we compared *D*_ACC→AIC_ with 0. If *D*_ACC→AIC_ >0, it indicated that the information flow was from ACC to AIC, and if *D*_ACC→AIC_ <0, it reflected that the information was from AIC to ACC.

We selected one bipolar channel (from two adjacent contacts) for a region of interest. If there were more than two channels within the same brain area, we chose the channel with no artifacts and a higher amplitude. For exploratory purposes, we also computed *h* ^2^ on raw signals filtered in seven EEG subbands, as mentioned above. The *h* ^2^ was performed using the following parameters: 2 s sliding window steps 1 s, and a maximum lag of 0.1 s using the Any‐Wave open‐source software (available at http://meg.univamu.fr/wiki/AnyWave).^24^ Further analysis was performed using MATLAB.

### 2.5 The slope of the aperiodic component of the power spectrum density

The power spectrum density (PSD) slope was obtained by using FOOOF. The frequency range was from 1 to 80 Hz. The slope is equivalent to the negative exponent when measured in log–log space due to aperiodic activity having a 1/*f*‐like distribution with exponentially decreasing power across increasing frequencies.^25^ The E:I ratio could be estimated from the 1/*f* power slope. The steeper the slope is, the lower the E:I ratio.^18^ A more negative slope indicates that relatively more inhibition occurs in the underlying neuronal populations.^22^ The power spectrum, *P*, was modeled using three parameters, as shown in equation (3):

(3) $$P=L+\sum_{n\;=\;0}^{N}G_{n}$$

where *L* is the aperiodic “background” signal, with *N* total peaks extracted from the power spectrum, and Gaussians (*G*_*n*_) fitted to each peak. The peaks were iteratively fitted by Gaussians, as shown in equation (4):

(4) $$G_{n}=a*\exp\left(\frac{-{\left(F-c\right)}^{2}}{2w^{2}}\right)$$

with a amplitude, *c* center frequency, *w* the bandwidth of the Gaussian *G*, and *F*, the input frequencies. The aperiodic signal *L* was modeled by, as shown in equation (5):

(5) $$L=b-\log\left(k+F^{\chi }\right)$$

where *b* is the broadband offset, *x* is the slope, and *k* is the “knee” parameter, which was set to 0. The FOOOF model was fitted for the frequency range of 1–80 Hz.

### 2.6 Statistical analysis

To test whether there were significant differences within the *h* ^2^ values of the ACC and AIC over different periods, the calculation was divided into two steps. In the first step, the average value of *h* ^2^ was calculated for all signal pairs for each sliding window and each period of interest. In the second step, the connectivity was measured by averaging the *h* ^2^ values obtained from *h* ^2^_ACC→AIC_ and *h* ^2^_AIC→ACC_.

The periodic oscillations were grouped according to the center frequency at seizure onset. The narrow‐band *h* ^2^ and *D* of different periods were compared. We performed pairwise comparisons of *h* ^2^, *D*, and PSD slope for different periods (IID versus PI, IID versus SO, PI versus SO, SO versus SP). First, normality tests were performed. If all groups met the normality and the variances between the two groups were homogeneous, we used paired t test for comparisons. If one of the groups dose not meet the normality test, a nonparametric Wilcoxon matched‐pairs signed rank test was considered. A *p*‐value < 0.05 was considered significant. *p*‐Values were corrected with the original false discovery rate (FDR) method of Benjamini and Hochberg.

## 3 RESULTS

### 3.1 Clinical information

Twenty patients with medically refractory epilepsy were enrolled, 10 patients with anterior cingulate epilepsy, and 10 patients with anterior insular epilepsy. The mean age at epilepsy onset was 13.3 years (range 2–30), and the mean age at SEEG was 19.4 years (range 2–41). The mean duration of epilepsy before SEEG was 6.1 years (range 0–13). Five patients had visible lesions on MRI (25%) and 15 were MRI‐negative (75%). Thirteen patients underwent surgical resection, and seven patients received RF‐TC. The surgical pathologies of 13 cases were diagnosed as follows: focal cortical dysplasia (FCD) type I in three cases, FCD type II in seven cases, and non‐specific in three cases. The surgical outcomes were Engel class I for 15 patients and Engel class II for 5 patients. The median follow‐up duration after surgery was 3.64 years (ranging from 1.17 to 7). Patient characteristics are summarized in Table 1 (Table S1).

**TABLE 1:** Patient clinical information.

Patient, clinical features	Number
Gender, female/male	7/13
Median age at epilepsy onset, years(min–max)	13.3(2–30)
Median age at SEEG, years(min–max)	19.4(2–41)
Median epilepsy duration, years (min–max)	6.1 (0–13)
Visible MRI lesion, *n* (%)	5 (25)
Epileptogenic zone, ACC/AIC	10/10
Treatment, resection/RF‐TC	13/7
Histological type	
FCD I	3
FCD II	7
Non‐special	3
Engel classes (%)	
I	15 (75)
II	5 (25)
Median follow‐up post‐SEEG (years, min‐max)	3.64 (1.17–7)

Abbreviations: ACC, anterior cingulate cortex; AIC, anterior insula cortex; FCD, focal cortical dysplasia. MRI, magnetic resonance imaging; SEEG, stereoelectroencephalography; RF‐TC, radiofrequency thermocoagulation.

### 3.2 SEEG onset analysis

In this study, visual and time‐frequency analyses were performed on 20 clinical seizures. The seizure‐onset pattern was low voltage fast activity (LVFA) in seven cases, sharp activity in four cases, and rhythmic spikes/poly spikes/spike waves in nine cases. The frequency of seizure onset was grouped as follows (Figure S1 and Table S2): six cases in the α band, six cases in the β1 band, five cases in the β2 band, two cases in the γ1 band, and one case in the γ2 band.

### 3.3 Functional connectivity based on the narrow‐band frequency

#### 3.3.1 Non‐linear correlation coefficient (*h* ^2^)

**FIGURE 2:**
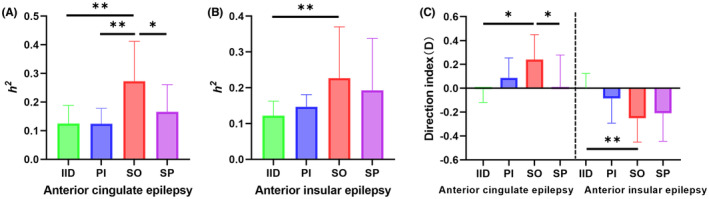
Functional connectivity based on the narrow‐band frequency. (A) The non‐linear correlation coefficient (*h* ^2^) between anterior cingulate cortex (ACC) and anterior insula cortex (AIC) during four periods in anterior cingulate epilepsy. The connectivity during seizure onset was significantly higher than that during other periods (*p < 0.05, **p < 0.01, paired *t*‐test). (B) *h* ^2^ between ACC and AIC during periods in anterior insular epilepsy. The connectivity during seizure onset was significantly higher than that during interictal (**p < 0.01, Wilcoxon test). (C) The direction index (*D*) in anterior cingulate epilepsy and anterior insular epilepsy during four periods. The four columns on the left side were compared in anterior cingulate epilepsy. The direction index (*D*) at seizure onset was significantly higher than that at interictal and seizure propagation (*p < 0.05, Wilcoxon test). The four columns on the right side were compared in anterior insular epilepsy. The direction index (*D*) at seizure onset was significantly higher than that at interictal (**p < 0.01, paired *t*‐test). IID, interictal; PI, preictal; SO, seizure onset; SP, seizure propagation.

In anterior cingulate epilepsy, the FC between ACC and AIC during seizure onset (SO) was significantly higher than that during IID (*p* = 0.0078), pre‐ictal (PI) (*p* = 0.0019), and seizure propagation (SP) (*p* = 0.0222) (Figure 2A). In anterior insular epilepsy, there was a significant difference between *h* ^2^ of IID and SO (*p* = 0.0098) (Figure 2B). The *h* ^2^ at seizure onset increased significantly, suggesting a hyperconnectivity between the ACC and AIC during this period.

#### 3.3.2 Direction index (D)

**FIGURE 3:**
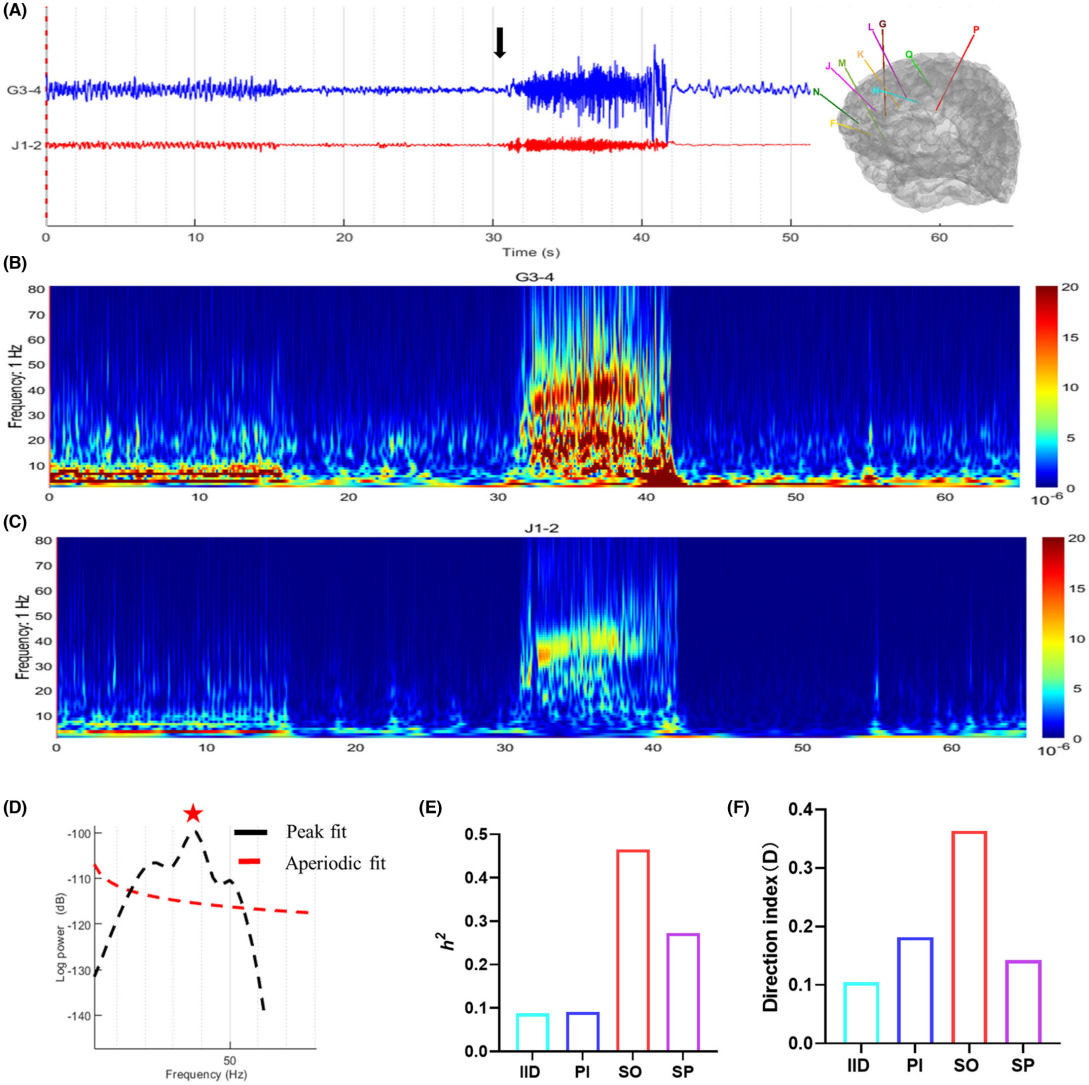
Example of patient 1 with anterior cingulate epilepsy. (A) Channel J1‐2 was located in the left anterior cingulate cortex (the epileptogenic zone), while G3‐4 was in the left anterior insular cortex (the propagation zone). They discharged almost simultaneously at seizure onset (black arrow). (B) Time‐frequency analysis of channel G3‐4 (anterior insular cortex, AIC). (C) Time‐frequency analysis of channel J1‐2 (anterior cingulate cortex, ACC). (D) Power spectral density of channel J1‐2 at seizure onset. The center frequency was 37.59 Hz. (E) The non‐linear correlation coefficient (*h* ^2^) between ACC and AIC increased significantly at seizure onset. (F) The direction index (*D*) between ACC and AIC during four periods was positive, suggesting that the information flow was from ACC to AIC.

**FIGURE 4:**
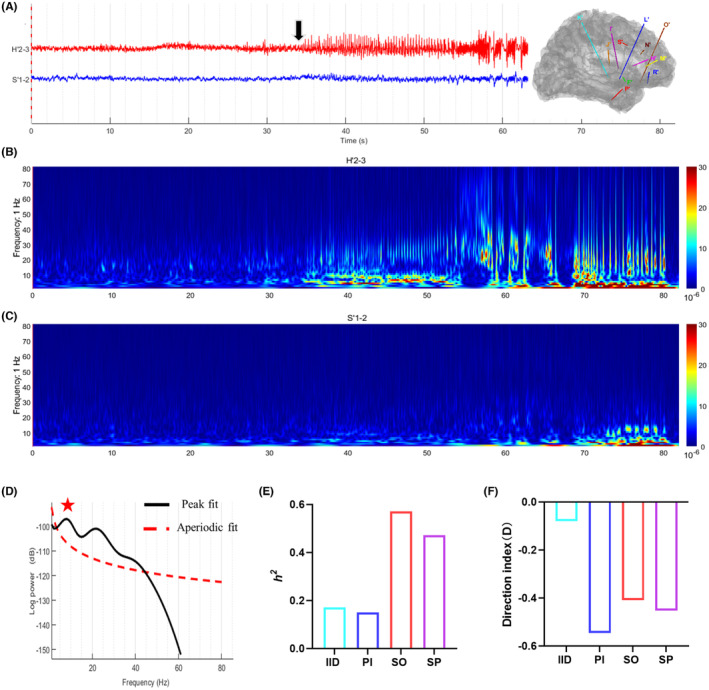
Example of patient 18 with anterior insular epilepsy. (A) Channel H′2–3 was located in the right anterior insular cortex (the epileptogenic zone) and S′1–2 was in the right anterior cingulate cortex (the propagation zone). Channel H′2–3 discharged at seizure onset (black arrow). (B) Time‐frequency analysis of channel H′2–3 (anterior insular cortex, AIC). (C) Time‐frequency analysis of channel S′1–2 (anterior cingulate cortex, ACC). (D) Power spectral density of channel H′2–3 at seizure onset. The center frequency was 8.51 Hz. (E) The non‐linear correlation coefficient (*h* ^2^) between ACC and AIC increased significantly at seizure onset. (F) The direction index (*D*) between ACC and AIC during four periods was negative, suggesting that the information flow was from AIC to ACC.

In both types of epilepsy, the direction index (D) of SO was significantly higher than that of IID (*p* = 0.0371 in anterior cingulate epilepsy and 0.0009 in anterior insular epilepsy, respectively; Figure 2C). In anterior cingulate epilepsy, the direction index (*D*) during PI and SO was positive, indicating that the connectivity was from ACC to AIC. In anterior insular epilepsy, the direction index (D) during PI, SO, and SP was negative, suggesting that the information flow was from AIC to ACC. The diagnostic accuracy of direction index (D) was 90% (9/10) in both types of epilepsy (Table S3). The direction index (*D*) could be used to identify the leader–follower relationship between ACC and AIC. Figures 3 and 4 illustrated two examples, patient 1 with anterior cingulate epilepsy and patient 18 with anterior insular epilepsy.

### 3.4 Excitation/Inhibition ratio revealed by the PSD slope

#### 3.4.1 The dynamic evolution of the E:I ratio during seizures

In the 10 cases of anterior cingulate epilepsy, the SOZ was identified as the ACC, while the non‐SOZ was located in the AIC. Similarly, in the other 10 cases of anterior insular epilepsy, the SOZ was considered as the AIC and non‐SOZ as the ACC. In SOZ, the E:I ratio of SO was significantly different than that of IID and PI (*p* < 0.0001 and *p* = 0.0008, respectively), suggesting hyperexcitability at seizure onset. The E:I ratio during PI increased significantly compared with that during IID (*p* = 0.0127). In non‐SOZ, the E:I ratio of SO was significantly higher than that of IID and PI (*p* = 0.0005 and *p* = 0.0056, respectively).

**FIGURE 5:**
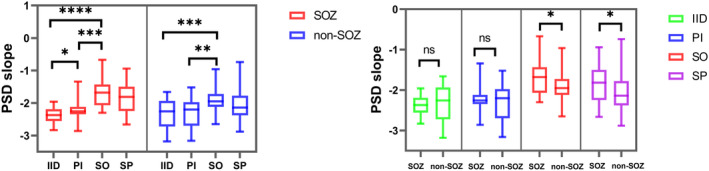
Excitation–Inhibition ratio revealed by the PSD slope. (A) Excitation/Inhibition ratio (E:I ratio) of the SOZ (red) and non‐SOZ (blue) during four periods. In SOZ, the PSD slope of seizure onset was significantly higher than that of interictal and preictal. In non‐SOZ, there is a similar trend at seizure onset (*p < 0.05, **p < 0.01, ***p < 0.001, ****p < 0.0001, paired *t*‐test). (B) The E:I ratio between SOZ and non‐SOZ during four periods. There was a significant difference in the E:I ratio between SOZ and non‐SOZ during seizure onset and seizure propagation (*p < 0.05, paired *t*‐test). IID, interictal; PI, preictal; SO, seizure onset; SP, seizure propagation.

The difference of E:I ratio between SOZ and non‐SOZ was significant in SO and SP periods (*p* = 0.0121 and *p* = 0.0364, respectively) (Figure 5B), suggesting that the excitability of SOZ changed dynamically at seizure onset. The results might assist in distinguishing SOZ and non‐SOZ (Table S4).

#### 3.4.2 The interaction between ACC and AIC during seizures

**FIGURE 6:**
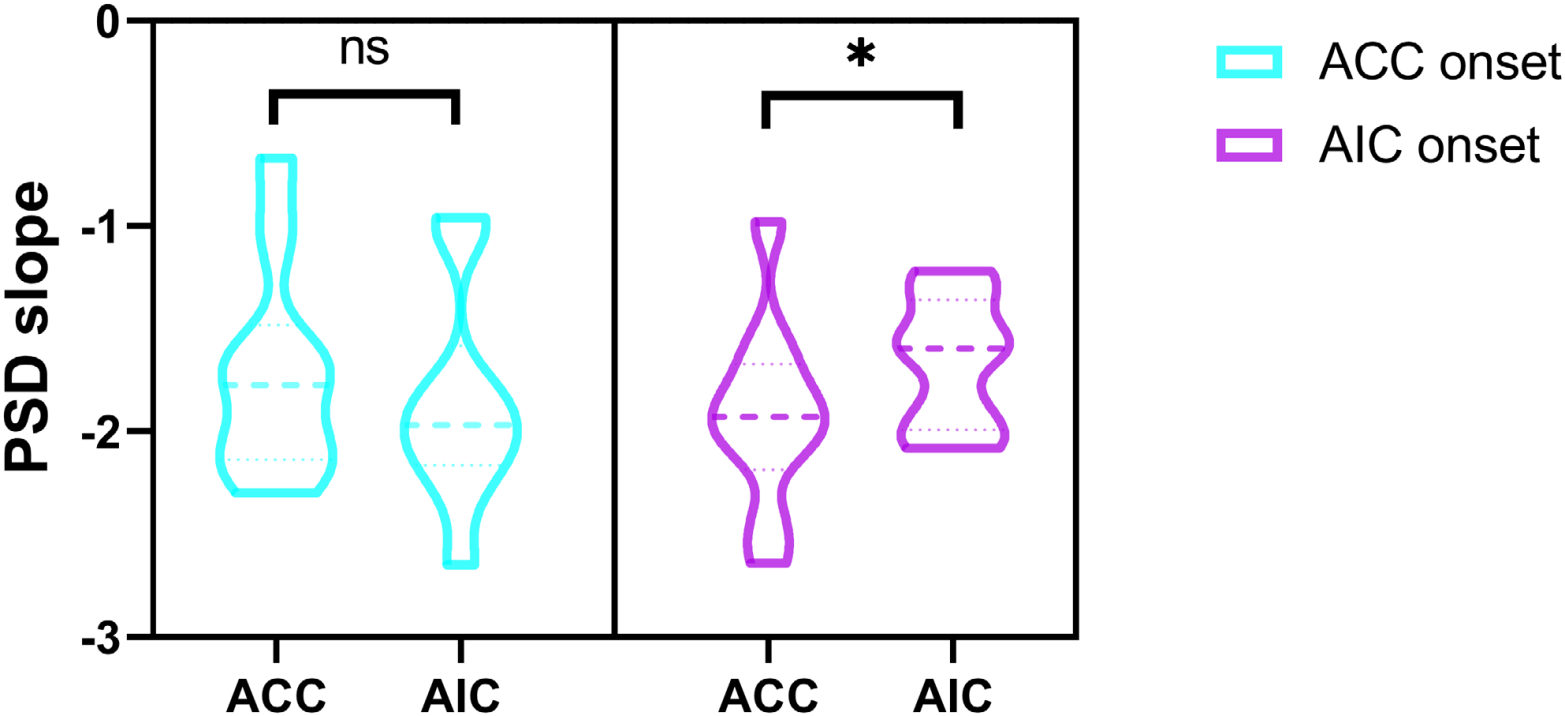
Excitation/Inhibition ratio at seizure onset. The E:I ratio of ACC and AIC at seizure onset (SO) in anterior cingulate epilepsy (left) and anterior insular epilepsy (right). There was no significant difference in the E:I ratio between ACC and AIC in anterior cingulate epilepsy (*p* > 0.05). There was a significant difference in the E:I ratio between ACC and AIC in anterior insular epilepsy (*p* = 0.0364). IID, interictal; PI, preictal; SO, seizure onset; SP, seizure propagation.

At seizure onset, there was no significant difference in the E:I ratio between ACC and AIC for seizures with onset in the ACC (*p* > 0.05) (Figure 6 left). However, for those originating from AIC, the E:I ratio was significantly higher in the AIC than in the ACC (*p* = 0.0364) (Figure 6 right). These findings suggest that the ACC and AIC might not exert equal influence on each other in excitability.

## 4 DISCUSSION

In the present study, we tried to elucidate two issues: (1) whether there exits FC between ACC and AIC during seizures; (2) how the ACC and AIC interact during seizure‐onset periods. To identify the SOZ in ACC and AIC, we tried to investigate: (1) whether there is a leader–follower relationship in FC between the SOZ and non‐SOZ during seizures; (2) how the excitability of both SOZ and non‐SOZ changes during seizures. We analyzed the interaction between ACC and AIC in two ways: connectivity and excitability. The non‐linear correlation coefficient (*h* ^2^), which indicates the strength of FC, increased significantly at seizure onset, suggesting that there exited functional connectivity between the ACC and AIC. The direction index (*D*) clarified the information flow between the ACC and AIC in two types of epilepsy, indicating that there was a leader–follower relationship between the SOZ and non‐SOZ during seizures. Temporally, the E:I ratio changed dynamically during seizures from interictal periods to ictal periods in both SOZ and non‐SOZ. Spatially, the excitability of SOZ changed more dynamically at seizure onset. Comparing ACC and AIC as SOZ, respectively, it was observed that there two brain regions did not influence each other in a similar way.

The EN becomes unstable due to perturbations between excitatory and inhibitory connections within a neural network.^26^ An imbalance of inhibitory and excitatory networks might alter connection strength between brain regions that are important nodes of the EN. With the present study, we speculated that the anterior cingulate‐insula network (aCIN) might be a potential EN that needs attention and further study.

### 4.1 Coactivation has a cytological and structural basis

The ACC and AIC share a unique type of cell in layer V, Von Economo Neurons (VENs),^4^ which might be the cytological basis for the coactivation of the ACC and AIC. The VENs are larger neurons with single, long, apical, and basal dendrites, suggesting faster transmission of information between regions and possibly more connections.^4^, ^27^ The activity of VENs has been related to error monitoring and evaluation of unexpected stimuli.^5^ The ACC, one of the areas with the highest density of VENs, integrates specialized networks for attentional processes/executive functions with sensory, high‐order associative, and limbic brain areas.^28^ The AIC, another area with a high density of VENs, is active in highly uncertain situations and saliency evaluation.^29^

Cytoarchitectonics studies have revealed that the insula can be divided into a rostroventral agranular zone, a transitional dysgranular zone, and a dorsocaudal granular zone.^6^ The AIC is an agranular region with the absence of an internal granular layer (IV). Similarly, the cingulate cortex has three divisions: an agranular ACC, a dysgranular midcingulate cortex (MCC), and a granular posterior cingulate cortex/retrosplenial cortex (PCC/RSC).^7^

The distribution of structural connectivity is related to its heterogeneous cytoarchitecture,^30^ and cortico‐cortical connections are densest among areas with comparable architecture. Macaque studies used tracer techniques to identify structural connections from the insula to the cingulate cortex.^31^, ^32^ In a study of rodents, a bidirectional connection between the ACC and AIC was observed.^33^ Many prior structural studies in humans had not revealed connections between the AIC and ACC until Ghaziri and colleagues demonstrated an anterior–posterior organization in the structural connections between the insula and cingulate cortex, and identified direct connectivity between the AIC and ACC.^8^

The ACC and AIC coactivate during many behaviors. The ACC and AIC receive dual lamina I spin‐thalamocortical projection and are coactivated in virtually all studies of emotion, so they are regarded as limbic sensory and motor cortices.^34^ The ACC evolved first as a motor control brain region, while the AIC evolved later for cortical processing of individual sensory activity, and these two regions became linked for integrative autonomic control.^35^ The AIC was considered to engender human awareness through close integration with the ACC.^36^ The ACC and AIC form the salience network that functions to segregate the most relevant among internal and extrapersonal stimuli to guide behavior.^29^ Within the salience network, the AIC acts as an integral hub and activates ACC to respond when salient events are detected.^37^ The causal signaling between the AIC and ACC plays a fundamental role in implementing cognitive control; the AIC first detects events requiring greater access to cognitive control resources and then signals the ACC to execute load‐specific cognitive control processes.^38^

#### 4.1.1 Functional connectivity between ACC and AIC in resting state

The concept of functional connectivity (FC) emphasizes the temporal correlation between distinct brain regions that share functional properties.^39^ The resting‐state fMRI study by Cauda et al. confirmed that the AIC connected to ACC functionally.^40^ These two brain regions are implicated in the functioning of the limbic system, and are involved in attentional processes, working memory, and higher‐order control processes. In the study be Deen et al., the human insular was parcellated based on clustering of functional connectivity patterns.^41^ They found that the ventral AIC exhibited the strongest correlation with pregenual ACC, whereas the dorsal AIC was functionally connected to dorsal ACC. The study conducted by Oane et al. utilizing combined electrophysiological methods failed to confirm a strong connection between ACC and AIC.^42^ This is consistent with our findings that the FC between ACC and AIC was weak during the interictal phase of seizures.

#### 4.1.2 Functional connectivity between ACC and AIC during seizures

A variety of methods have been utilized to investigate FC, of which SEEG stands out for its high temporal resolution. The non‐linear correlation coefficient has been shown to reflect the strength of FC between different brain regions.^43^ A study by Hagiwara et al. confirmed a significant directed coupling from insula to cingulate gyrus during seizures in patients with insular lobe epilepsy.^44^ Their results demonstrated that the insula could drive the cingulate regions, which signifies that the cingulate propagation is common in the insular lobe epilepsy. In the present study, our results showed a significant enhancement in the strength of connection between ACC and AIC at seizure onset, indicating an elevated level of synchronization between these two regions.

The non‐linear nonparametric regression also calculated the direction index (*D*) to determine the direction of information flow,^13^ which can help to identify that leader–follower relationships do exist between the SOZ and non‐SOZ during seizures. In some patients with drug‐resistant epilepsy, the ACC and AIC exhibit synchronous onset on SEEG due to early propagation, making visual distinction challenging. By utilizing the direction index (*D*), we can make an informed decision prior to surgery.

There is an increasing trend in coupling between ACC and AIC with the progression of the epileptic process. However, the interpretation for the increase in synchronization at seizure onset is challenging due to its inherent complexity. For two non‐identical coupled non‐linear systems, Rulkov et al. defined the term generalized synchronization.^45^ In order to assess the degree of synchrony, we employ time lag *t* to estimate the temporal delay between two signals from ACC and AIC. Our results showed that despite an increase in synchronization, there exist temporal lags and a driving response mechanism between ACC and AIC. Yet, it should be noted that the results above are statistical phenomena and further research is required wo elucidate the underlying electrophysiological mechanisms.

#### 4.1.3 Dynamic interaction between ACC and AIC during seizures

Electrophysiological signals have both periodic and aperiodic components. The periodic oscillations have been widely studied, yet the aperiodic component has long been ignored or is treated as noise. The aperiodic component form part of the PSD, which will always estimate nonzero power, even there is no detectable oscillation present.^22^ In the electrophysiological signal data, the distribution of aperiodic activity is characterized similarly to the function *y* = 1/*f* ^*x*^, with an exponential decrease in PSD with increasing frequency. The *x* parameter is equivalent to the negative slope of the PSD when measured in log–log space.^46^ Under physiological conditions, synaptic excitation and inhibition are maintained in a balanced state temporally and spatially.^47^ The E/I balance is essential for neuronal homeostasis and formation of neural oscillations^48^, ^49^ and is crucial for effective information transmission, maintenance of working memory.^50^, ^51^ Conversely, E/I imbalance may lead to numbers of neuropsychiatric disorders, such as epilepsy.^52^

Given such a state of intricate balance and its profound consequences when disturbed, quantifying the E:I ratio could aid in better characterizing the dynamic evolution during seizures. The computation model developed by Gao et al. suggested that the E:I ratio can be quantified from the PSD, with a flatter PSD slope indicating a higher E:I ratio.^18^ We found a significant change in the power slope between the interictal and ictal periods, with a flatter slope at seizure onset. While in the findings of Jiang et al.^53^ which indicated that the power slope in the SOZ was more negative than that in the non‐SOZ in the resting state. There are two possible reasons which might explain this disagreement: (1) the different brain regions of interest examined (anterior cingulate epilepsy and anterior insular epilepsy versus temporal lobe epilepsy); (2) The different frequency ranges selected (1–80 Hz vs. 1–250 Hz). The power slope, as an indicator of the E:I ratio, deserves more extensive study in the future.

The semiology of epilepsy depends on the dynamic interaction between the anatomical origins of ictal discharges and the propagation zone within existing neural pathways.^54^ Therefore, the clinical semiology of a given seizure assists in identifying the neural network involved in epileptic electrical activity. The “chapeau de gendarme” (CDG) sign is considered a semiological marker of the anterior cingulate‐insula network.^55^ The occurrence of CDG can be related to the simultaneous involvement of a network including the ACC and AIC.^56^ The mechanism of CDG might be related to a cortico‐subcortical network, and the ACC and AIC were found to be involved, suggesting reciprocal connectivity between these two areas in the context of epilepsy.

There are limitations in the interpretation of these results. A relatively small cohort of patients was analyzed, we may not exclude the influence of clinical variables. The sampling rate of SEEG recording was 512 or 256 Hz, and the data were analyzed from 1 to 80 Hz. High‐frequency oscillations above 80 Hz were not included, which might have a potential influence on the results. Moreover, the slope of the aperiodic component of the power spectrum is an indirect measure of the E:I ratio and has not been extensively validated.

## 5 CONCLUSION

Coactivation is a specific pattern of ACC and AIC interaction in physiological situations. In the context of epilepsy, these two brain regions are dynamically coupled during seizures. The functional connectivity between ACC and AIC increased significantly at seizure onset. The excitability changed dynamically during seizures temporally and spatially. The direction index (*D*) can be used to identify the source of information flow between SOZ and non‐SOZ. The excitability of SOZ changes more significantly than that of non‐SOZ. The ACC and AIC might not exert equal influence on each other in excitability.

## AUTHOR CONTRIBUTIONS

TL, MW, and GL contributed to the conception and design of the study. All authors contributed to the acquisition and analysis of data. YY contributed to drafting the test and preparing the figures. DC contributed to interpreting the results. All authors reviewed and revised the manuscript for intellectual content.

## CONFLICT OF INTEREST STATEMENT

The authors declared no conflict of interest.

## Supporting information


Data S1.

## Data Availability

The data that support the findings of this study are available from the corresponding author upon reasonable request.
