# Supplementary material for: Dynamic evolution of the anterior cingulate‐insula network during seizures
Source: CNS Neurosci Ther. 2023 Jun 12;29(12):3901–12. doi: 10.1111/cns.14310 (PMC10651990; doi:10.1111/cns.14310)
Supplement: Supplementary file 1 — Data S1. [file CNS-29-3901-s001.doc]

Supplementary Table 1. Main clinical features of the 20 studied patients.

| Pt/No. | Gender | Handness | Age at onset (years) | Age at  SEEG (years) | EZ | Scalp-IEDs  (lateral/location) |
| --- | --- | --- | --- | --- | --- | --- |
| 1 | M | R | 12 | 20 | ACC | L/F |
| 2 | F | R | 10 | 16 | ACC | Diffused |
| 3 | F | R | 6 | 13 | ACC | L/F-C |
| 4 | M | R | 24 | 24.5 | ACC | L/F |
| 5 | M | R | 12 | 13 | ACC | L/F |
| 6 | F | R | 4 | 5 | ACC | Bi/F |
| 7 | F | R | 2 | 2 | ACC | none |
| 8 | M | R | 10 | 17 | ACC | Diffused |
| 9 | M | R | 15 | 24 | ACC | L/F |
| 10 | M | Bi | 24 | 25 | ACC | L/F-T |
| 11 | M | R | 23 | 23 | AIC | L/F |
| 12 | F | L | 6 | 14 | AIC | L/F-C |
| 13 | M | R | 30 | 41 | AIC | L/T-F |
| 14 | M | R | 10 | 23 | AIC | Diffused |
| 15 | M | Bi | 3 | 7 | AIC | R/F-C |
| 16 | F | R | 27 | 32 | AIC | L/F |
| 17 | M | R | 15 | 16 | AIC | L/F-C |
| 18 | M | R | 12 | 19 | AIC | R/hemispheric |
| 19 | F | R | 3 | 8 | AIC | R/F |
| 20 | M | R | 4 | 11 | AIC | R/T-P |

(Next page)

Abbreviations: M = male; F = female; R = right; L = left; Bi = bilateral; SEEG = stereoelectroencephalography; EZ = epileptogenic zone; IEDs = interictal epileptiform discharges; ACC = anterior cingulate cortex; AIC = anterior insular cortex; F = frontal lobe; T = temporal lobe; F-C = frontocentral region; F-T = frontotemporal region; T-P = temporoparietal region.

Diffused is similar to generalized, but less symmetrical than generalized.

(continued)

| Pt/No. | Semiology recorded on scalp-ictal EEG |
| --- | --- |
| 1 | The chapeau de gendarme sign → autonomic seizure → right arm tonic → complex motor seizure |
| 2 | Axial tonic → right arm tonic → left deviation →complex motor seizure |
| 3 | Autonomic seizure → complex motor seizure |
| 4 | Hypermotor seizure |
| 5 | The chapeau de gendarme sign → right arm tonic →autonomic seizure |
| 6 | Axial tonic → the chapeau de gendarme sign → hypermotor seizure |
| 7 | Autonomic seizure → complex motor seizure |
| 8 | Autonomic aura → axial tonic → autonomic seizure |
| 9 | Autonomic aura → the chapeau de gendarme sign → hypermotor seizure |
| 10 | Autonomic aura → complex motor seizure → GTCS |
| 11 | Complex motor seizure |
| 12 | Bilateral asymmetric tonic → complex seizure |
| 13 | Automotor seizure → right arm hypomotor → left deviation → right versive → GTCS |
| 14 | Axial tonic → left deviation → right arm tonic → GTCS |
| 15 | Fear aura → complex motor seizure |
| 16 | Fear aura → autonomic seizure |
| 17 | Autonomic seizure → hypomotor → right deviation → GTCS |
| 18 | The chapeau de gendarme sign → hypermotor seizure |
| 19 | Somatosensory aura → axial tonic → left leg hypomotor |
| 20 | Nonspecific aura → automatic seizure→ automotor → left versive → left tonic-clonic seizure |

(Next page)

Abbreviations: GTCS = generalized tonic-clonic seizure. The semiology is based on the classification of paroxysmal events and a four-dimensional epilepsy classification system ^1^.

(continued)

| Pt/No. | Scalp-ictal EEG onset (lateral / location) | MRI lesion  (lateral / location) | MEG  (lateral / location) | PET hypometabolism  (lateral / location) |
| --- | --- | --- | --- | --- |
| 1 | Bi / FT | Normal | L / F | L /P and T |
| 2 | Diffused | L / mesial F | - | L /F and T |
| 3 | Diffused | L / ACC | L / F | - |
| 4 | Bi / F | Normal | negative | L / T |
| 5 | L / F | Normal | L / F | L /F and P |
| 6 | Bi / F | L / mesial F | - | L / mesial F |
| 7 | Diffused | Normal | - | - |
| 8 | Diffused | R / choroid fissure cyst | R / F and Oper | - |
| 9 | L / F | Normal | - | L / T |
| 10 | L / F | Normal | negative | R / mesial T |
| 11 | L / F | Normal | L / F and C | L / hemispheric |
| 12 | L / FC | Normal | C | - |
| 13 | Diffused | Normal | L / T | L / T |
| 14 | Diffused | Normal | L /F and Ins | L /mesial T |
| 15 | Diffused | Normal | negative | L / T |
| 16 | L / F | Bi/PWM | negative | L / F |
| 17 | Diffused | Normal | - | L / hemispheric |
| 18 | Diffused | Normal | - | R/Ins |
| 19 | R / F | Normal | R / F | L /P and T |
| 20 | Diffused | Normal | R / C | R / Oper |

(Next page)

Abbreviations: PWM = paraventricular white matter; Oper = operculum; Ins = insula; P = parietal lobe;

(continued)

| Pt/No. | No. of  electrodes | Treatment | Histological type | Follow-up  （months） | Outcome  (Engel) |
| --- | --- | --- | --- | --- | --- |
| 1 | 10 | Resection | FCD Ⅱ b | 74 | Ⅰ |
| 2 | 7 | Resection | FCD Ⅰ b | 63 | Ⅰ |
| 3 | 10 | Resection | FCD Ⅱ b | 39 | Ⅰ |
| 4 | 16 | Resection | FCD Ⅱ a | 31 | Ⅰ |
| 5 | 11 | RF-TC | - | 14 | Ⅰ |
| 6 | 5 | Resection | FCD Ⅱ b | 65 | Ⅰ |
| 7 | 15 | RF-TC | - | 71 | Ⅱ |
| 8 | 15 | Resection | not special | 55 | Ⅰ |
| 9 | 11 | Resection | FCD Ⅱ a | 84 | Ⅰ |
| 10 | 11 | RF-TC | - | 24 | Ⅰ |
| 11 | 7 | RF-TC | - | 46 | Ⅰ |
| 12 | 12 | Resection | FCD Ⅱ b | 31 | Ⅱ |
| 13 | 15 | RF-TC | - | 34 | Ⅱ |
| 14 | 15 | RF-TC | - | 32 | Ⅱ |
| 15 | 14 | Resection | FCD Ⅰ b | 22 | 1 |
| 16 | 13 | RF-TC | - | 31 | Ⅱ |
| 17 | 10 | Resection | not special | 28 | Ⅰ |
| 18 | 12 | Resection | FCD Ⅰ b | 30 | Ⅰ |
| 19 | 10 | Resection | FCD Ⅱ b | 45 | Ⅰ |
| 20 | 12 | Resection | not special | 55 | Ⅰ |

Abbreviations: RF-TC = radiofrequency thermocoagulation; FCD = focal cortical dysplasia.

Supplementary Table 2. SEEG onset analysis

| Pt/No. | SOP | EZ | Channel of interest | Periodic components of SO | | | Band group |
| --- | --- | --- | --- | --- | --- | --- | --- |
|  |  |  |  | center-frequency (Hz) | amplitude | std_dev (Hz) |  |
| 1 | LVFA | ACC | J1-2 | 37.59 | 1.54 | 4.58 | γ1 |
| 2 | DC shift→Sharp activity | ACC | J3-4 | 9.93 | 1.12 | 2.96 | α |
| 3 | LVFA | ACC | H3-4 | 28.53 | 1.49 | 2.47 | β2 |
| 4 | Sharp beta activity | ACC | L1-2 | 24.23 | 0.60 | 6.00 | β2 |
| 5 | Rhythmic spikes | ACC | H1-2 | 13.00 | 1.38 | 4.22 | β1 |
| 6 | Rhythmic polyspikes | ACC | K2-3 | 22.32 | 1.14 | 5.98 | β2 |
| 7 | LVFA | ACC | H'1-2 | 55.35 | 0.77 | 5.60 | γ2 |
| 8 | LVFA | ACC | X'2-3 | 29.36 | 0.85 | 4.32 | β2 |
| 9 | Rhythmic spikes | ACC | M3-4 | 13.66 | 0.47 | 3.95 | β1 |
| 10 | Rhythmic spike-waves | ACC | H1-2 | 10.04 | 0.88 | 3.48 | α |
| 11 | LVFA | AIC | J4-5 | 26.66 | 1.29 | 6.00 | β2 |
| 12 | Rhythmic spike-waves | AIC | N5-6 | 14.52 | 1.65 | 6.00 | β1 |
| 13 | Sharp alpha activity | AIC | M1-2 | 8.01 | 1.41 | 3.31 | α |
| 14 | Burst of polyspikes | AIC | M6-7 | 34.32 | 0.54 | 5.99 | γ1 |
| 15 | Rhythmic spikes | AIC | K'2-3 | 13.10 | 0.71 | 4.45 | α |
| 16 | LVFA | AIC | H2-3 | 19.85 | 0.55 | 3.11 | β1 |
| 17 | LVFA | AIC | J2-3 | 17.26 | 1.11 | 5.99 | β1 |
| 18 | Rhythmic spikes | AIC | H’2-3 | 8.51 | 0.97 | 3.34 | α |
| 19 | Rhythmic spikes | AIC | J'2-3 | 19.79 | 1.11 | 6.00 | β1 |
| 20 | Sharp theta activity | AIC | J'2-3 | 9.32 | 1.21 | 1.47 | α |

Abbreviations: ACC = anterior cingulate cortex; AIC = anterior insula cortex; DC shift = direct current shift; LVFA = low voltage fast activity; SO = seizure onset; SOP = seizure onset pattern.

Supplementary Table 3. The correlation coefficient *h^2^* and direction index D during four periods.

| Pt/No. | *h^2^* | D |
| --- | --- | --- |

|  | IID | | PI | SO | | SP | |  | | IID | PI | | | SO | | SP |
| --- | --- | --- | --- | --- | --- | --- | --- | --- | --- | --- | --- | --- | --- | --- | --- | --- |
| 1 | 0.09 | 0.11 | | 0.44 | 0.07 | |  | | 0.10 | | | 0.18 | 0.31 | | -0.09 | |
| 2 | 0.27 | 0.24 | | 0.44 | 0.34 | |  | | 0.07 | | | 0.21 | 0.33 | | -0.12 | |
| 3 | 0.07 | 0.16 | | 0.48 | 0.20 | |  | | -0.10 | | | 0.02 | 0.08 | | 0.28 | |
| 4 | 0.07 | 0.08 | | 0.24 | 0.22 | |  | | 0.04 | | | 0.15 | -0.25 | | -0.54 | |
| 5 | 0.12 | 0.16 | | 0.23 | 0.14 | |  | | -0.24 | | | -0.14 | 0.48 | | -0.03 | |
| 6 | 0.16 | 0.12 | | 0.21 | 0.15 | |  | | -0.02 | | | -0.15 | 0.43 | | 0.49 | |
| 7 | 0.05 | 0.04 | | 0.04 | 0.03 | |  | | -0.09 | | | 0.14 | 0.31 | | -0.02 | |
| 8 | 0.13 | 0.09 | | 0.21 | 0.11 | |  | | -0.03 | | | 0.37 | 0.18 | | 0.05 | |
| 9 | 0.16 | 0.12 | | 0.17 | 0.12 | |  | | 0.11 | | | 0.15 | 0.34 | | 0.11 | |
| 10 | 0.13 | 0.12 | | 0.27 | 0.28 | |  | | 0.09 | | | -0.05 | 0.20 | | -0.03 | |
| 11 | 0.11 | 0.15 | | 0.17 | 0.08 | |  | | 0.06 | | | 0.05 | -0.09 | | -0.17 | |
| 12 | 0.09 | 0.14 | | 0.12 | 0.18 | |  | | 0.21 | | | 0.08 | -0.12 | | 0.18 | |
| 13 | 0.14 | 0.16 | | 0.39 | 0.53 | |  | | -0.11 | | | 0.11 | -0.43 | | -0.24 | |
| 14 | 0.08 | 0.15 | | 0.17 | 0.09 | |  | | 0.03 | | | 0.01 | -0.40 | | -0.01 | |
| 15 | 0.21 | 0.16 | | 0.19 | 0.14 | |  | | -0.18 | | | 0.04 | -0.53 | | -0.31 | |
| 16 | 0.09 | 0.11 | | 0.26 | 0.09 | |  | | -0.02 | | | -0.11 | -0.38 | | -0.45 | |
| 17 | 0.12 | 0.22 | | 0.13 | 0.12 | |  | | -0.02 | | | 0.00 | 0.09 | | -0.45 | |
| 18 | 0.17 | 0.16 | | 0.56 | 0.37 | |  | | -0.13 | | | -0.56 | -0.39 | | -0.54 | |
| 19 | 0.11 | 0.10 | | 0.17 | 0.19 | |  | | -0.01 | | | -0.20 | -0.10 | | -0.15 | |
| 20 | 0.10 | 0.12 | | 0.11 | 0.14 | |  | | 0.17 | | | -0.27 | -0.18 | | 0.05 | |

Abbreviations: *h^2^* = nonlinear correlation coefficient; D = direction index; IID = interictal; PI = pre-ictal; SO = seizure onset; SP = seizure propagation.

Supplementary Table 4. The PSD slope during four periods.

| Pt/No. | | Selected | | | PSD slope | | | | | | | |
| --- | --- | --- | --- | --- | --- | --- | --- | --- | --- | --- | --- | --- |
|  | channel | | | IID | | | PI | | SO | | SP | |
|  | EZ | | Non-EZ | EZ | | Non-EZ | EZ | Non-EZ | EZ | Non-EZ | EZ | Non-EZ |
| 1 | J1-2 | | G3-4 | -2.48 | | -2.24 | -2.42 | -2.06 | -0.67 | -0.96 | -1.61 | -2.24 |
| 2 | J3-4 | | M6-7 | -2.74 | | -2.94 | -2.86 | -2.99 | -2.07 | -1.84 | -2.66 | -2.20 |
| 3 | H3-4 | | J2-3 | -2.24 | | -1.75 | -2.18 | -2.02 | -1.04 | -1.02 | -0.97 | -0.74 |
| 4 | L1-2 | | J2-3 | -2.19 | | -1.66 | -2.22 | -1.52 | -2.30 | -1.98 | -2.57 | -2.58 |
| 5 | H1-2 | | J1-2 | -2.09 | | -2.43 | -2.35 | -2.20 | -2.18 | -1.96 | -2.18 | -1.77 |
| 6 | K2-3 | | J1-2 | -2.33 | | -3.18 | -2.10 | -2.72 | -2.12 | -2.65 | -1.66 | -2.88 |
| 7 | H'1-2 | | J'2-3 | -2.41 | | -2.78 | -1.96 | -1.95 | -1.63 | -2.12 | -1.50 | -2.60 |
| 8 | X'2-3 | | Y'2-3 | -1.96 | | -2.55 | -1.42 | -1.99 | -1.68 | -2.11 | -1.87 | -2.10 |
| 9 | M3-4 | | E2-3 | -2.64 | | -2.46 | -2.27 | -2.29 | -1.68 | -1.77 | -0.94 | -1.46 |
| 10 | H1-2 | | J1-2 | -2.04 | | -2.08 | -2.32 | -2.70 | -1.87 | -2.30 | -1.96 | -2.32 |
| 11 | J4-5 | | M1-2 | -2.54 | | -1.90 | -2.26 | -2.69 | -1.22 | -0.98 | -2.30 | -1.57 |
| 12 | N5-6 | | P2-3 | -2.12 | | -1.98 | -2.54 | -2.43 | -2.08 | -1.83 | -1.98 | -2.35 |
| 13 | M1-2 | | Q2-3 | -2.25 | | -2.81 | -1.34 | -2.84 | -1.85 | -2.10 | -1.96 | -2.18 |
| 14 | M6-7 | | L2-3 | -2.83 | | -3.04 | -2.48 | -3.16 | -1.97 | -2.64 | -2.27 | -2.39 |
| 15 | K'2-3 | | L'1-2 | -2.35 | | -2.48 | -2.33 | -2.53 | -1.56 | -2.03 | -2.52 | -2.50 |
| 16 | H2-3 | | L1-2 | -2.54 | | -1.89 | -2.25 | -2.01 | -2.06 | -2.45 | -1.50 | -1.96 |
| 17 | J2-3 | | H1-2 | -2.78 | | -2.20 | -2.17 | -1.97 | -1.39 | -1.71 | -1.51 | -1.86 |
| 18 | H’2-3 | | S'1-2 | -2.20 | | -2.27 | -1.52 | -1.53 | -1.59 | -1.56 | -1.12 | -1.60 |
| 19 | J'2-3 | | L'1-2 | -2.56 | | -2.10 | -2.32 | -2.20 | -1.27 | -1.92 | -1.36 | -1.99 |
| 20 | J'2-3 | | K'2-3 | -2.39 | | -1.92 | -2.18 | -1.87 | -1.60 | -1.94 | -1.76 | -1.80 |

Abbreviations: EZ = epileptogenic zone; PZ = propagation zone; IID = interictal; PI = pre-ictal; SO = seizure onset; SP = seizure propagation

References

1. Luders H, Vaca GF, Akamatsu N, et al. Classification of paroxysmal events and the four-dimensional epilepsy classification system*. Epileptic Diso*rd. Feb 1 2019;21(1):1-29. doi:10.1684/epd.2019.1033

Supplementary Figure 1 Examples of seizure onset pattern and time-frequency analysis


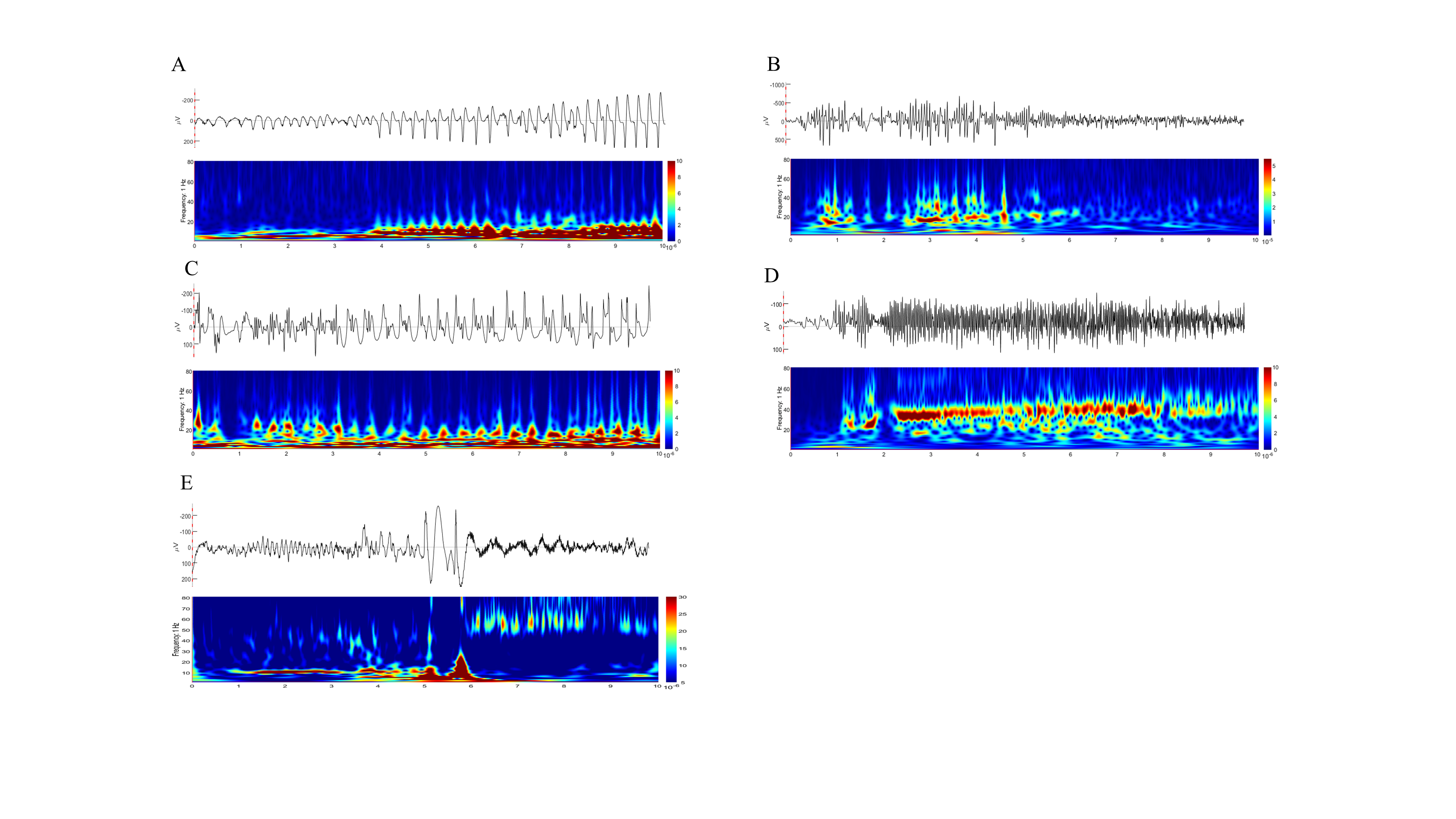
Legend: A. Sharp activity in the alpha range (8-13 Hz); B. Rhythmic spikes in the low beta range (13-20 Hz); C. Rhythmic polyspikes in the high beta range (20-30 Hz); D. Low-voltage fast activity (LVFA) in the low gamma range (30-45 Hz); E. Preictal spiking followed by LVFA in the high gamma range (45-80 Hz).
